# Associations of abnormal blood lipid levels with hospitalization cost and disease severity in patients with hyperlipidemic pancreatitis: a retrospective cohort study

**DOI:** 10.1186/s12876-026-05143-3

**Published:** 2026-07-21

**Authors:** Wenna Xu, Jichen Wang, Yang Zong, Qiang Fu, Ping Liu, Dao’an Ma, Kun Li, Yee Chao, Zhenxing Zeng, Min Wang, Minxuan Hu, Guozhong Ji

**Affiliations:** 1 Department of Gastroenterology, The Second Affiliated Hospital of Nanjing Medical University, Nanjing, Jiangsu China; 2 Department of Gastroenterology, Jen Ching Memorial Hospital, Suzhou, Jiangsu China; 3 Department of General Practice, The Second Affiliated Hospital of Nanjing Medical University, Nanjing, Jiangsu China

**Keywords:** Hyperlipidemic pancreatitis, Triglycerides, Total cholesterol, Hospitalization cost, Disease severity, Healthcare resource utilization

## Abstract

**Background:**

Hyperlipidemic pancreatitis (HLP) carries a considerable clinical and economic burden. This retrospective study examined how abnormal blood lipid levels relate to hospitalization cost and disease severity in patients with HLP, with the aim of informing risk stratification and lipid management in clinical practice.

**Methods:**

We retrospectively reviewed 117 patients with HLP accounting for 171 hospitalizations at Jen Ching Memorial Hospital between January 2018 and June 2025. Total hospitalization cost (THC) was analyzed by gamma regression and length of stay (LOS) by negative binomial regression, with cluster-robust standard errors to account for repeated hospitalizations in the same patient.

**Results:**

At the first hospitalization, the median triglyceride (TG) level was 13.56 mmol/L and the median total cholesterol (TC) level was 8.89 mmol/L. The median LOS was 6 days and the median THC was 10,920.44 yuan (US$1,528.85). In multivariable analyses, both lipids were positively associated with THC. Each 1 mmol/L increase in TG corresponded to a 2.2% higher THC (approximately 240 yuan; US$33.60) (rate ratio [RR] = 1.022, 95% confidence interval [CI] = 1.008–1.040, *P* = 0.013). Each 1 mmol/L increase in TC corresponded to a 5.3% higher THC (approximately 580 yuan; US$81.20) (RR = 1.053, 95% CI = 1.004–1.098, *P* = 0.046). Higher TG levels were further associated with greater odds of intensive care unit (ICU) transfer and complications.

**Conclusion:**

Higher TG and TC levels were associated with greater hospitalization cost, and higher TG levels with severity-related indicators. These associations suggest that lipid levels may be useful for risk stratification in patients with HLP.

**Supplementary Information:**

The online version contains supplementary material available at https://doi.org/10.1186/s12876-026-05143-3.

## Introduction

Acute pancreatitis (AP) is a common gastrointestinal disorder, with a rising global incidence of approximately 13–48 per 100,000 population per year [1–3]. Although biliary and alcoholic pancreatitis predominated in the past, hyperlipidemic pancreatitis (HLP) has become a growing concern and is now recognized as the second leading cause of AP in China, surpassing alcoholic pancreatitis [4, 5]. HLP presents a clinical challenge to the healthcare system due to its insidious onset, diagnostic complexity, tendency to affect younger patients, greater severity, and high recurrence rate, all of which may contribute to a substantial healthcare burden [6].

The pathophysiology of HLP is linked to triglyceride (TG)-driven microcirculatory injury, free fatty acid toxicity, and inflammatory cascades, and emerging evidence suggests that total cholesterol (TC) is also related to HLP progression and severity [7, 8]. However, current studies on HLP have largely focused on epidemiological patterns and treatment approaches, and few studies have examined healthcare resource utilization and economic outcomes in HLP. In particular, evidence regarding factors associated with disease-specific cost and disease severity in HLP remains insufficient.

Using clinical data from patients treated at Jen Ching Memorial Hospital between January 2018 and June 2025, this study aimed to analyze how abnormal blood lipid levels relate to hospitalization cost and disease severity in patients with HLP and to discuss their relevance to clinical risk assessment and management. These findings may help identify patients with HLP who are likely to incur higher costs and inform lipid-based risk assessment.

## Materials and methods

### Study population

We retrospectively reviewed 117 patients with HLP, accounting for 171 hospitalizations, treated at Jen Ching Memorial Hospital between January 2018 and June 2025. Inclusion criteria: (1) patients meeting the diagnostic criteria of Chinese Guidelines for the Diagnosis and Treatment of Acute Pancreatitis (2021); (2) those with TG ≥ 11.3 mmol/L (1000 mg/dL), or TG 5.65–11.3 mmol/L (500–1000 mg/dL) and chylous serum; (3) those with complete medical records; (4) those with an age of 18–70 years. Exclusion criteria: (1) patients with other definite etiologies such as gallstones, alcohol, drugs, or toxins; (2) those with chronic pancreatitis or pancreatic tumor; (3) those being observed in the emergency department and not hospitalized.

A total of 117 patients with 171 hospitalization records (mean 1.46 per person) were included in this study. To account for within-patient correlation due to repeated hospitalizations, hospitalization records were used as the primary unit of analysis, and all regression analyses used cluster-robust standard errors clustered by patient identification numbers (patient ID). Laboratory, imaging, and treatment variables were taken from each hospitalization record of the patient. For reporting baseline characteristics, the variables from each patient’s first hospitalization record were used as the primary data source.

### Definitions of variables

AP was diagnosed according to the *Chinese Guidelines for the Diagnosis and Treatment of Acute Pancreatitis* (2021) [6]. Type 2 diabetes mellitus (T2DM) was defined according to *Guideline for the Prevention and Treatment of Type 2 Diabetes Mellitus in China* (2020) [9]. Disease severity was assessed using the modified computed tomography (CT) severity index (MCTSI), which is the sum of scores for pancreatic/peripancreatic inflammatory changes, extent of pancreatic necrosis, and extra-pancreatic complications [10].

All laboratory variables such as TG, TC, low-density lipoprotein cholesterol (LDL-C), high-density lipoprotein cholesterol (HDL-C), and serum amylase, as well as the imaging findings were obtained within 24 h of admission.

Smoking history was defined as current or former regular use of any smoked *tobacco* product (≥ 100 cigarettes in a person’s lifetime). Alcohol use history was defined as consumption of an average of ≥ 14 standard drinks per week in men or ≥ 7 standard drinks per week in women for at least 6 months. History of hyperlipidemia was defined as previously documented TG ≥ 1.7 mmol/L, TC ≥ 5.2 mmol/L, LDL-C ≥ 3.4 mmol/L, or previous diagnosis of hyperlipidemia with or without lipid-lowering medication.

Complications included local complications, such as pancreatic necrosis and pseudocyst, and systemic complications, including systemic inflammatory response syndrome (SIRS), sepsis, respiratory failure, renal failure, circulatory failure, and multiple organ dysfunction syndrome (MODS).

Total hospitalization cost (THC) included all direct medical expenses during the hospitalization period, encompassing bed charges and costs for all nursing care, medication, laboratory and diagnostic tests, therapeutic interventions, surgical procedures, and consumables. All costs were adjusted for inflation using China’s consumer price index (CPI) and reported in 2025 Chinese yuan. For international readers, costs were additionally converted to US dollars using the 2025 annual average exchange rate of 1 USD = 7.1429 CNY, calculated based on the RMB central parity rates published by the China Foreign Exchange Trade System (CFETS). Length of stay (LOS) was defined as the total number of days from the date of admission to the date of discharge.

### Study variables

Baseline data included demographics (age, sex, and body mass index [BMI]), laboratory variables obtained within 24 h of admission (TG, TC, LDL-C, HDL-C, and serum amylase), and relevant medical history (T2DM, smoking, alcohol use, and hyperlipidemia). The economic endpoints were THC, reported in both Chinese yuan and US dollars, and LOS.

### Data collection and quality control

Cost data were extracted from the hospital information system (HIS) and matched one-to-one with electronic medical records. Data were standardized and checked for outliers using boxplot in R software (version 4.4.2). Outliers were detected using Tukey’s outer fences, with thresholds defined as values below the first quartile (Q1) minus 3 times the interquartile range (IQR), or above the third quartile (Q3) plus 3 times the IQR.

### Statistical analysis

Continuous variables were summarized as medians with IQR and categorical variables as counts and percentages (n, %). Collinearity was assessed with Spearman’s rank correlation coefficient (r) and variance inflation factors (VIFs).

LOS and MCTSI were analyzed using negative binomial regression and reported as incidence rate ratios (IRRs). THC was analyzed using gamma regression with a log link and reported as rate ratios (RRs). Complications and ICU transfer were analyzed using Firth-corrected logistic regression and reported as odds ratios (ORs). Because 117 patients contributed 171 hospitalization records, all regression analyses were performed using cluster-robust standard errors clustered by patient ID. The exponentiated coefficients (IRRs/RRs) with 95% confidence intervals (CIs) were displayed in a forest plot to aid interpretation. Two-sided *P* < 0.05 was considered statistically significant.

### Ethics statement

Because this was a retrospective study, all data were anonymized and de-identified before analysis, thereby ensuring that no individuals would be identified. The study protocol had been approved by the Ethics Committee of Jen Ching Memorial Hospital. The requirement for informed consent was waived due to the retrospective nature of this study and the use of anonymized data. This study was conducted in accordance with the ethical principles of the 2024 Declaration of Helsinki.

## Results

### Outlier detection

Using Tukey’s outer fences (thresholds: Q1–3 × IQR and Q3 + 3 × IQR), a small number of outliers were detected in five variables: serum amylase 1 (0.6%), LOS 2 (1.2%), THC 7 (4.2%), HDL-C 2 (1.2%), LDL-C 3 (1.8%). No outlier values beyond thresholds were found for age, TC, TG, MCTSI, and BMI. All manually reviewed outliers were clinically plausible values rather than recording errors and thus were retained for analysis.

### Patient characteristics

Baseline characteristics were taken from each patient’s first admission. Of the 117 patients, 80.3% (94/117) were men and 19.7% (23/117) were women; the median age was 38 years (IQR 32–44) and the median BMI was 27.4 kg/m² (IQR 25.2–29.4). On admission, the median TC was 8.89 mmol/L (IQR 6.63–11.75), TG 13.56 mmol/L (IQR 8.81–18.01), LDL-C 3.81 mmol/L (IQR 2.99–5.26), HDL-C 1.57 mmol/L (IQR 1.10–2.98), and serum amylase 160 U/L (IQR 85–402). The median MCTSI on imaging was 2 (IQR 2–4). The median LOS was 6 days (IQR 5–8), and the median THC was 10,920.44 yuan (US$1,528.85; IQR 8,508.45–14,521.61 yuan [US$1,191.18–2,033.01]). During the first hospitalization, 47.0% (55/117) developed complications and 10.3% (12/117) required ICU transfer.

### Collinearity assessment

Among the lipid measures (LDL-C, HDL-C, TC, and TG), Spearman correlation coefficients ranged from 0.41 to 0.72 (all *P* < 0.001). The strongest pairings were TC with HDL-C (*r* = 0.72), TC with LDL-C (*r* = 0.70), and TC with TG (*r* = 0.67), all below the prespecified threshold of 0.80. The corresponding VIFs were 1.76, 3.64, 4.73, and 1.68 for LDL-C, HDL-C, TC, and TG; because none exceeded 5, severe multicollinearity was not a concern in the models.

### Assessment of factors associated with LOS and THC using negative binomial and Gamma models

With within-patient correlation handled through cluster-robust standard errors clustered by patient ID, the lipid levels showed the strongest associations with the economic outcomes. The remaining covariates—age, sex, BMI, LDL-C, HDL-C, serum amylase, T2DM, smoking, and alcohol history—were not significantly associated with LOS or THC, their 95% CIs all crossing 1 (Tables 1 and 2).

**Table 1:** Adjusted IRRs for variables associated with LOS in the negative binomial model

Variable	Model	IRR	95% CI	*P* value
Age (year)	LOS (IRR)	0.996	0.988–1.004	0.259
Sex	LOS (IRR)	1.093	0.794–1.511	0.545
BMI (kg/m²)	LOS (IRR)	1.002	0.972–1.028	0.963
TG (mmol/L)	LOS (IRR)	1.018	1.003–1.028	0.029^*^
T2DM	LOS (IRR)	1.064	0.894–1.262	0.457
History of hyperlipidemia	LOS (IRR)	0.953	0.813–1.110	0.433
TC (mmol/L)	LOS (IRR)	1.041	1.014–1.081	0.015^*^
Serum amylase (U/L)	LOS (IRR)	1.000	1.000–1.000	0.305
Smoking history	LOS (IRR)	0.928	0.769–1.100	0.293
Alcohol use history	LOS (IRR)	1.033	0.872–1.229	0.704
HDL-C (mmol/L)	LOS (IRR)	0.976	0.894–1.037	0.327
LDL-C (mmol/L)	LOS (IRR)	1.002	0.973–1.027	0.993

**Table 2:** Adjusted RRs for variables associated with hospitalization cost in the Gamma-log model

Variable	Model	RR	95% CI	*P* value
Age (year)	THC (RR)	0.994	0.983–1.004	0.315
Sex	THC (RR)	1.032	0.732–1.449	0.852
BMI (kg/m²)	THC (RR)	1.015	0.981–1.042	0.391
TG (mmol/L)	THC (RR)	1.022	1.008–1.040	0.013^*^
T2DM	THC (RR)	0.974	0.801–1.163	0.672
History of hyperlipidemia	THC (RR)	1.081	0.856–1.372	0.440
TC (mmol/L)	THC (RR)	1.053	1.004–1.098	0.046^*^
Serum amylase (U/L)	THC (RR)	1.000	1.000–1.000	0.307
Smoking history	THC (RR)	0.885	0.732–1.061	0.146
Alcohol use history	THC (RR)	1.023	0.844–1.251	0.811
HDL-C (mmol/L)	THC (RR)	0.972	0.839–1.132	0.692
LDL-C (mmol/L)	THC (RR)	1.003	0.966–1.037	0.943

Both TG and TC levels were positively associated with LOS and THC. Specifically, for TG, each 1 mmol/L higher TG level was independently associated with an estimated 1.8% longer LOS (IRR = 1.018, *P* = 0.029) and a 2.2% higher THC (RR = 1.022, *P* = 0.013). Based on the median THC (10,920.44 yuan), this coefficient corresponded to approximately 240 yuan (US$33.60) higher THC per 1 mmol/L higher TG level.

Notably, TC showed a larger per-unit association with THC than TG in this model. Each 1 mmol/L increase in TC was associated with a 4.1% longer LOS (IRR = 1.041, *P* = 0.015) and a 5.3% higher THC (RR = 1.053, *P* = 0.046), corresponding to approximately 580 yuan (US$81.20).

To illustrate the model-based associations, we generated estimates of LOS and THC across clinically relevant lipid ranges, and used the median TG (13.56 mmol/L) and TC (8.89 mmol/L) as reference levels (Tables 3 and 4).

The model-based estimates suggested a substantial cost difference between patients with different TG levels. For example, the estimated THC differed by approximately 4,442 yuan (US$621.88) between a relatively lower TG level of 6 mmol/L and severe hypertriglyceridemia of 24 mmol/L, ranging from 9,263.85 yuan (US$1,296.93) to 13,705.90 yuan (US$1,918.81), corresponding to an estimated 48.0% higher cost (Table 3).

**Table 3:** Model-based estimates of LOS and THC across different TG levels

TG (mmol/L)	Change in LOS (%)	Estimated LOS (days)	Change in THC (%)	Estimated THC (yuan)	Estimated THC (US$)
6.00	-12.62%	5.24	-15.17%	9,263.85	1,296.93
8.00	-9.44%	5.43	-11.40%	9,675.94	1,354.62
10.00	-6.15%	5.63	-7.45%	10,106.36	1,414.88
12.00	-2.74%	5.84	-3.34%	10,555.94	1,477.82
13.56*	0.00%	6.00	0.00%	10,920.44	1,528.85
14.00	+ 0.79%	6.05	+ 0.96%	11,025.51	1,543.56
16.00	+ 4.45%	6.27	+ 5.45%	11,515.96	1,612.23
18.00	+ 8.24%	6.49	+ 10.14%	12,028.24	1,683.94
20.00	+ 12.17%	6.73	+ 15.04%	12,563.31	1,758.85
22.00	+ 16.25%	6.97	+ 20.16%	13,122.17	1,837.09
24.00	+ 20.47%	7.23	+ 25.51%	13,705.90	1,918.81

*indicates the median TG level at baseline. Estimates were derived from the negative binomial model for LOS and the Gamma-log model for THC using the median TG level as the reference. Costs are presented in 2025 Chinese yuan. US dollar equivalents were calculated using the exchange rate of 1 USD = 7.1429 CNY

Similarly, when TC varied from 6 mmol/L to 14 mmol/L, the model-estimated THC ranged from 9,406.37 yuan (US$1,316.88) to 14,218.35 yuan (US$1,990.56), corresponding to an estimated 51.2% higher cost (Table 4).

**Table 4:** Model-based estimates of LOS and THC across different TC levels

TC (mmol/L)	Change in LOS (%)	Estimated LOS (days)	Change in THC (%)	Estimated THC (yuan)	Estimated THC (US$)
6.00	-10.96%	5.34	-13.86%	9,406.37	1,316.88
8.00	-3.51%	5.79	-4.49%	10,429.87	1,460.17
8.89*	0.00%	6.00	0.00%	10,920.44	1,528.85
10.00	+ 4.56%	6.27	+ 5.90%	11,564.73	1,619.05
12.00	+ 13.31%	6.80	+ 17.42%	12,823.08	1,795.22
14.00	+ 22.79%	7.37	+ 30.20%	14,218.35	1,990.56

* indicates the median TC level at baseline. Estimates were derived from the negative binomial model for LOS and the Gamma-log model for THC using the median TC level as the reference. Costs are presented in 2025 Chinese yuan. US dollar equivalents were calculated using the exchange rate of 1 USD = 7.1429 CNY

### Forest plot

**Fig. 1:**
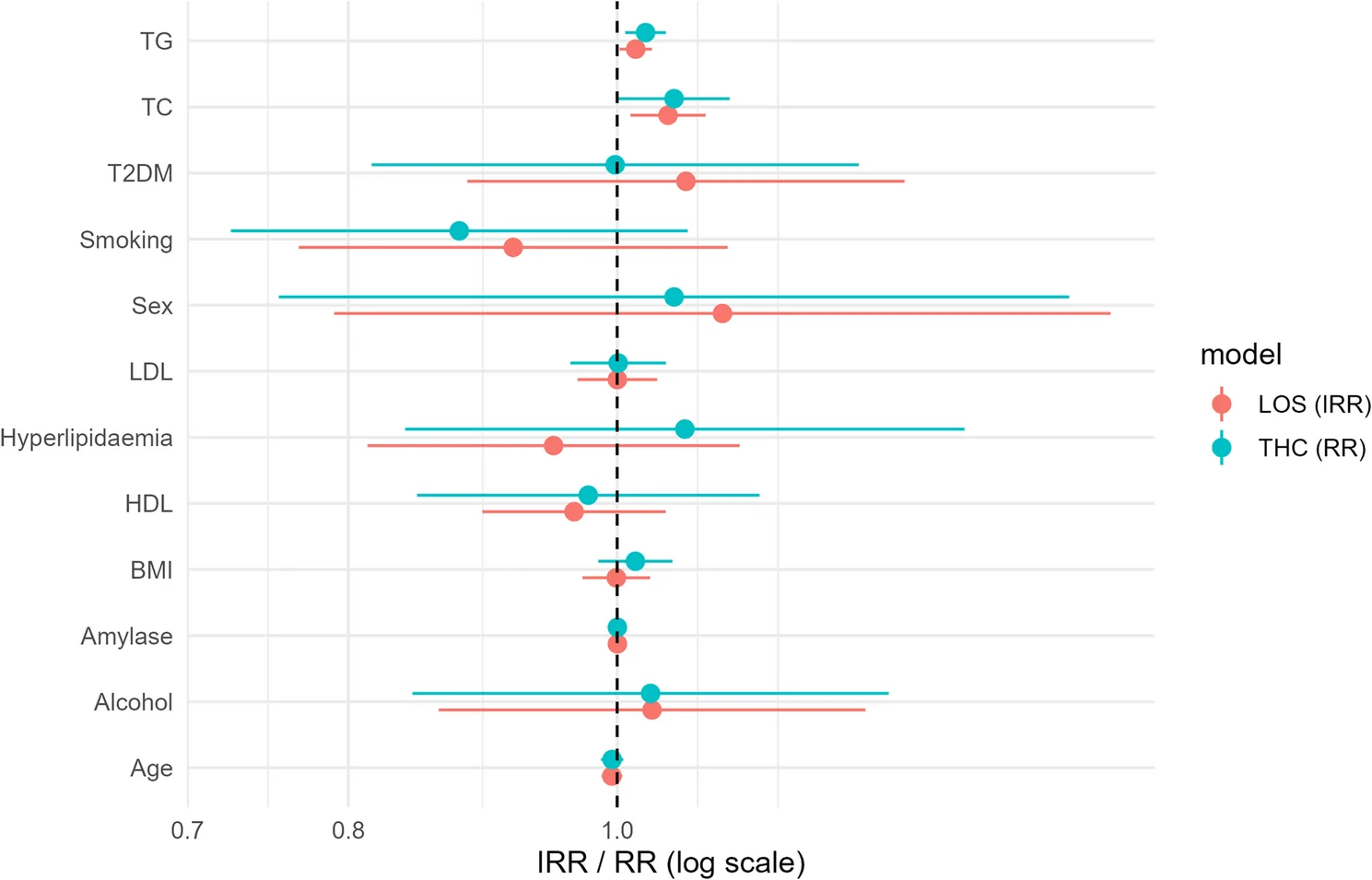
Forest plot of multivariable regression analysis results. Forest plot displaying the adjusted IRR for LOS (red points) and RR for THC (blue points). Horizontal lines represent 95% CIs. Abbreviations: TG, triglycerides; TC, total cholesterol; T2DM, type 2 diabetes mellitus; LDL-C, low-density lipoprotein cholesterol; HDL-C, high-density lipoprotein cholesterol; BMI, body mass index; IRR, incidence rate ratio; RR, rate ratio; LOS, length of stay; THC, total hospitalization cost

The forest plot (Fig. 1) illustrated the results of multivariable analysis: the red points denoted IRR for LOS, the blue points denoted RR for THC. The point estimates and 95% CIs of both TG and TC were to the right of the null line, indicating that both TG and TC showed significant positive associations with LOS and THC. Other variables showed no significant associations, as their 95% CIs crossed the null line.

### Logistic regression analysis of complications and ICU transfer

We analyzed complications and ICU transfer in patients with HLP using Firth-corrected logistic regression with cluster-robust standard errors clustered by patient ID. The results indicated that TG level was independently associated with higher odds of ICU transfer (OR = 1.19, 95% CI = 1.094–1.291, *P* < 0.001) and complications (OR = 1.11, 95% CI = 1.027–1.204, *P* = 0.007). Other variables were not significantly associated with complications or ICU transfer.

### Negative binomial regression analysis for MCTSI

The negative binomial regression analysis of MCTSI in patients with HLP indicated that a higher TG level was significantly associated with an increased MCTSI (IRR = 1.04, 95% CI = 1.026–1.050, *P* < 0.001). Other covariates (TC, age, sex, BMI, T2DM, history of hyperlipidemia, serum amylase, smoking history, alcohol use history, HDL-C, LDL-C) were not significantly associated with MCTSI. These findings suggest that higher TG levels were associated with severity-related clinical indicators.

## Discussion

This study provides an economic perspective on HLP by quantifying the associations between abnormal blood lipid levels and hospitalization cost. In this single-center retrospective cohort, higher TG and TC levels were associated with longer LOS and higher THC after accounting for within-patient correlation due to repeated hospitalizations. Admission lipid levels, particularly TG, may help identify patients with greater disease burden and higher resource use.

The association between TG levels and higher hospitalization cost may be partly explained by disease severity. In our cohort, higher TG levels were associated with higher MCTSI, higher odds of ICU transfer, and higher odds of complications. These findings suggest that patients with higher TG levels may have more severe clinical presentations, which could contribute to greater healthcare resource utilization.

In addition to our primary findings, we conducted a sensitivity analysis to explore the potential threshold patterns by categorizing TG and TC levels, as shown in Supplementary Table S1. The results indicated that neither the medium nor high TG groups, nor the higher TC group, exhibited statistically significant differences in THC or LOS compared with the reference group (all *P* > 0.05). This suggests that the association between lipid levels and economic burden may be continuous rather than threshold-dependent. Overall, higher lipid levels may help identify patients with higher expected healthcare resource utilization.

Previous studies have indicated an increased AP risk at TG > 11.3 mmol/L and a markedly increased severe AP risk at TG > 22.6 mmol/L, with high TG levels linked to MODS and pancreatic necrosis [11–13]. A recent study also reported that higher TC levels were associated with more severe imaging findings, higher Acute Physiology and Chronic Health Evaluation II (APACHE II) scores and mortality [14]. Clinical and database analyses have also shown that HLP is associated with longer LOS and higher THC, and greater use of high-cost therapies such as blood purification [15]. An analysis of data from the US Nationwide Inpatient Sample (NIS) further indicated that, compared with non-HLP, HLP was associated with an average increase of US$1,672 in THC, representing an increase in cost by more than 10% [16]. These findings are consistent with the observed associations between lipid abnormalities, disease severity, and healthcare resource utilization in our cohort.

Although treatment strategies were not directly evaluated in this study, TG-lowering therapy remains clinically relevant in HLP management. In the acute phase of HLP, supportive care remains the foundation of treatment. Insulin infusion may be used to promote TG clearance, whereas therapeutic plasma exchange (TPE) may be considered in selected patients with HLP and severe hypertriglyceridemia, particularly when TG levels decline slowly with conservative treatment or in pregnancy; however, current evidence suggests only moderately faster TG reduction with apheresis and limited evidence for improved clinical outcomes compared with conservative therapy [17]. For long-term management of severe hypertriglyceridemia, dietary and lifestyle modification, control of secondary causes, and pharmacological TG-lowering therapy are important. Fibrates are generally recommended as first-line agents for patients at risk of HLP, while omega-3 fatty acids and niacin may be considered as adjunctive options in selected patients [18]. In patients with familial chylomicronemia syndrome or persistent chylomicronemia, conventional TG-lowering therapies may be insufficient, and apolipoprotein C-III (ApoC-III)-targeted therapies have emerged as potential options [19]. Recent phase 3 trials have suggested that ApoC-III-targeted therapies, including olezarsen and plozasiran, may reduce AP events in selected patients with familial chylomicronemia syndrome or persistent chylomicronemia [20, 21].

These findings have potential implications for risk stratification and follow-up management. Patients with markedly elevated TG or TC levels may represent a subgroup with higher expected healthcare resource utilization and may warrant closer lipid monitoring, dietary counseling, and post-discharge follow-up. This is particularly relevant because HLP has been associated with higher THC than other forms of AP, possibly related to prolonged treatment and resource-intensive interventions such as TPE and continuous renal replacement therapy (CRRT) [22]. Moreover, patients with HLP in China are predominantly young men with a median age of 40 years, who are considerably younger than patients with biliary or alcoholic AP [23], suggesting that complications may impose both direct medical costs and indirect socioeconomic burden. However, whether such risk-based follow-up strategies can reduce complications, LOS, or hospitalization cost should be evaluated in prospective studies.

Several limitations should be considered. First, this was a single-center retrospective observational study, and causal relationships between lipid levels, disease severity, and hospitalization cost cannot be established. Second, although cluster-robust standard errors were used to account for repeated hospitalizations by the same patient, residual confounding may still exist, and information on treatment adherence, post-discharge lipid control, and detailed treatment strategies was not fully captured. Third, although higher TG levels were associated with higher MCTSI, ICU transfer, and complications, we did not perform a formal mediation analysis to determine whether disease severity mediated the association between TG levels and hospitalization cost. In addition, severe acute pancreatitis was not analyzed as a separate categorical outcome; therefore, MCTSI, ICU transfer, and complications were used as severity-related clinical indicators. Future multicenter prospective studies are warranted to validate these findings and evaluate whether lipid-lowering strategies may improve clinical and economic outcomes.

## Conclusion

In patients with HLP, higher TG and TC levels were associated with greater hospitalization cost. Higher TG levels were also associated with MCTSI, complications, and ICU transfer. Admission lipid profiles may help with risk stratification and follow-up planning.

## Supplementary Information

Supplementary Material 1.

## Data Availability

The datasets generated and/or analysed during the current study are not publicly available due to patient privacy concerns but are available from the corresponding author on reasonable request.

## References

[1] Iannuzzi JP, King JA, Leong JH, Quan J, Windsor JW, Tanyingoh D, Coward S, Forbes N, Heitman SJ, Shaheen AA, et al. Global Incidence of Acute Pancreatitis Is Increasing Over Time: A Systematic Review and Meta-Analysis. Gastroenterology. 2022;162(1):122–34. 10.1053/j.gastro.2021.09.043 34571026

[2] Lin XY, Zeng Y, Zhang ZC, Lin ZH, Chen LC, Ye ZS. Incidence and clinical characteristics of hypertriglyceridemic acute pancreatitis: A retrospective single-center study. World J Gastroenterol. 2022;28(29):3946–59. 10.3748/wjg.v28.i29.3946 PMC9367230 36157550

[3] Reiche WS, Walters RW. Trends and Outcomes of US Hypertriglyceridemia-Induced Acute Pancreatitis Hospitalizations in Hispanic Americans: Epidemiology from 2016 to 2020. J Racial Ethn Health Disparities. 2025;12(6):3714–20. 10.1007/s40615-024-02171-1 PMC12644205 39251499

[4] Petrov MS, Yadav D. Global epidemiology and holistic prevention of pancreatitis. Nat Rev Gastroenterol Hepatol. 2019;16(3):175–84. 10.1038/s41575-018-0087-5 PMC6597260 30482911

[5] Qiu M, Zhou X, Zippi M, Goyal H, Basharat Z, Jagielski M, Hong W. Comprehensive review on the pathogenesis of hypertriglyceridaemia-associated acute pancreatitis. Ann Med. 2023;55(2):2265939. 10.1080/07853890.2023.2265939 PMC10563627 37813108

[6] Chinese Pancreatic Surgery Association CSoSCMA. [Guidelines for diagnosis and treatment of acute pancreatitis in China (2021)]. Zhonghua Wai Ke Za Zhi. 2021;59(7):578–87. 10.3760/cma.j.cn112139-20210416-00172 34256457

[7] Qiu M, Huang Y, Zhou X, Yu J, Li J, Wang W, Zippi M, Fiorino S, Hong W. Hyperlipidemia exacerbates acute pancreatitis via interactions between P38MAPK and oxidative stress. Cell Signal. 2025;125:111504. 10.1016/j.cellsig.2024.111504 39505288

[8] Kiss L, Fur G, Pisipati S, Rajalingamgari P, Ewald N, Singh V, Rakonczay Z Jr. Mechanisms linking hypertriglyceridemia to acute pancreatitis. Acta Physiol (Oxf). 2023;237(3):e13916. 10.1111/apha.13916 36599412

[9] Zhu D. Guideline for the prevention and treatment of type 2 diabetes mellitus in China (2020 edition). Chin J Diabetes Mellitus. 2021;13(4):315–409.

[10] Mortele KJ, Wiesner W, Intriere L, Shankar S, Zou KH, Kalantari BN, Perez A, vanSonnenberg E, Ros PR, Banks PA, et al. A modified CT severity index for evaluating acute pancreatitis: improved correlation with patient outcome. AJR Am J Roentgenol. 2004;183(5):1261–5. 10.2214/ajr.183.5.1831261 15505289

[11] Huang C, Liu J, Lu Y, Fan J, Wang X, Liu J, Zhang W, Zeng Y. Clinical features and treatment of hypertriglyceridemia-induced acute pancreatitis during pregnancy: A retrospective study. J Clin Apher. 2016;31(6):571–8. 10.1002/jca.21453 26946248

[12] Mosztbacher D, Hanak L, Farkas N, Szentesi A, Miko A, Bajor J, Sarlos P, Czimmer J, Vincze A, Hegyi PJ, et al. Hypertriglyceridemia-induced acute pancreatitis: A prospective, multicenter, international cohort analysis of 716 acute pancreatitis cases. Pancreatology. 2020;20(4):608–16. 10.1016/j.pan.2020.03.018 32402696

[13] Kim SJ, Kang H, Kim EJ, Kim YS, Cho JH. Clinical features and outcomes of hypertriglyceridemia-induced acute pancreatitis: Propensity score matching analysis from a prospective acute pancreatitis registry. Pancreatology. 2020;20(4):617–21. 10.1016/j.pan.2020.03.013 32265135

[14] Chen Z, Huang X, Han N, Guo Y, Chen J, Ning Y, Zhang M. Total cholesterol concentration predicts the effect of plasmapheresis on hypertriglyceridemic acute pancreatitis: a retrospective case-control study. BMC Gastroenterol. 2021;21(1):3. 10.1186/s12876-020-01572-w PMC7789165 33407166

[15] Reed JM, Hogan BM, Nasser-Ghodsi N, Loftus CG. Management of Hypertriglyceridemia-Induced Acute Pancreatitis in a Nondiabetic Patient. Mayo Clin Proc Innov Qual Outcomes. 2021;5(2):520–4. 10.1016/j.mayocpiqo.2020.08.015 PMC8105512 33997648

[16] Kleinman A, Kammerer J, Kleinman N, Desai NR. EE322 Cost and Clinical Characteristics of Acute Pancreatitis Hospitalizations Related and Unrelated to Hypertriglyceridemia. Value Health. 2025;28(6):S121.

[17] Gubensek J. The role of apheresis and insulin therapy in hypertriglyceridemic acute pancreatitis-a concise review. BMC Gastroenterol. 2023;23(1):341. 10.1186/s12876-023-02957-3 PMC10546782 37789261

[18] Berglund L, Brunzell JD, Goldberg AC, Goldberg IJ, Sacks F, Murad MH, Stalenhoef AF. Endocrine s: Evaluation and treatment of hypertriglyceridemia: an Endocrine Society clinical practice guideline. J Clin Endocrinol Metab. 2012;97(9):2969–89. 10.1210/jc.2011-3213 PMC3431581 22962670

[19] Javed F, Hegele RA, Garg A, Patni N, Gaudet D, Williams L, Khan M, Li Q, Ahmad Z. Familial chylomicronemia syndrome: An expert clinical review from the National Lipid Association. J Clin Lipidol. 2025;19(3):382–403. 10.1016/j.jacl.2025.03.013 40234111

[20] Watts GF, Rosenson RS, Hegele RA, Goldberg IJ, Gallo A, Mertens A, Baass A, Zhou R, Muhsin M, Hellawell J, et al. Plozasiran for Managing Persistent Chylomicronemia and Pancreatitis Risk. N Engl J Med. 2025;392(2):127–37. 10.1056/NEJMoa2409368 39225259

[21] Stroes ESG, Alexander VJ, Karwatowska-Prokopczuk E, Hegele RA, Arca M, Ballantyne CM, Soran H, Prohaska TA, Xia S, Ginsberg HN, et al. Olezarsen, Acute Pancreatitis, and Familial Chylomicronemia Syndrome. N Engl J Med. 2024;390(19):1781–92. 10.1056/NEJMoa2400201 38587247

[22] Kessler AS, Kutrieb E, Soffer D, Abramovitz L, Llonch MV, Moynahan A, Weycker D, Baum S. Healthcare costs among acute pancreatitis patients with severe hypertriglyceridemia. J Clin Lipidol. 2025;19(3):e118–9.

[23] Fan Z, Li J, Wu D. Hypertriglyceridemic pancreatitis: perspectives from China. Curr Opin Gastroenterol 2025. 10.1097/MOG.0000000000001123 PMC12337944 40682402

