# Supplementary material for: Associations of abnormal blood lipid levels with hospitalization cost and disease severity in patients with hyperlipidemic pancreatitis: a retrospective cohort study
Source: BMC Gastroenterol. 2026 Jul 21;26:649. doi: 10.1186/s12876-026-05143-3 (PMC13670667; doi:10.1186/s12876-026-05143-3)
Supplement: Supplementary file 1 [file 12876_2026_5143_MOESM1_ESM.docx]

To explore potential threshold patterns in the associations between lipid levels and economic burden, we conducted a sensitivity analysis by categorizing triglyceride (TG) and total cholesterol (TC) levels. TG was categorized into three clinically relevant groups: low TG, < 11.3 mmol/L; medium TG, 11.3–22.6 mmol/L; and high TG, > 22.6 mmol/L. TC was categorized into quartiles according to its distribution in the study cohort: Q1, ≤ 6.63 mmol/L; Q2, > 6.63 to ≤ 8.89 mmol/L; Q3, > 8.89 to ≤ 11.75 mmol/L; and Q4, > 11.75 mmol/L.

Length of stay (LOS) was analyzed using negative binomial regression, and total hospitalization cost (THC) was analyzed using Gamma regression with a log link. The models used the same covariate adjustment framework as the primary analysis, and cluster-robust standard errors were used to account for repeated hospitalizations within the same patient. The results are presented in Supplementary Table S1.

Table S1. Sensitivity analysis of categorized TG and TC levels in relation to LOS and THC

| **Outcome Variable** | **Predictor Variable** | **IRR / RR** | **95% CI** | ***P*** |
| --- | --- | --- | --- | --- |
| **Length of Stay (LOS)** |  | **IRR** |  |  |
| *(Model: Negative Binomial)* | **TG Group (Ref: < 11.3)** |  |  |  |
|  | Medium TG (11.3–22.6) | 0.963 | 0.779–1.189 | 0.723 |
|  | High TG (> 22.6) | 0.962 | 0.687–1.348 | 0.822 |
|  | **TC Group (Ref: Q1)** |  |  |  |
|  | Q2 | 0.855 | 0.655–1.117 | 0.251 |
|  | Q3 | 0.819 | 0.622–1.077 | 0.150 |
|  | Q4 | 0.863 | 0.656–1.134 | 0.286 |
|  |  |  |  |  |
| **Total Hospitalization Cost (THC)** |  | **RR** |  |  |
| *(Model: Gamma Regression)* | **TG Group (Ref: < 11.3)** |  |  |  |
|  | Medium TG (11.3–22.6) | 0.975 | 0.812–1.171 | 0.791 |
|  | High TG (> 22.6) | 0.917 | 0.683–1.231 | 0.562 |
|  | **TC Group (Ref: Q1)** |  |  |  |
|  | Q2 | 1.176 | 0.925–1.496 | 0.183 |
|  | Q3 | 1.074 | 0.842–1.370 | 0.561 |
|  | Q4 | 1.188 | 0.932–1.515 | 0.164 |

Notes: IRR, incidence rate ratio; RR, rate ratio; CI, confidence interval; LOS, length of stay; THC, total hospitalization cost; TG, triglyceride; TC, total cholesterol. IRR is reported for the LOS model, and RR is reported for the THC model. TG categories were defined as low TG, < 11.3 mmol/L; medium TG, 11.3–22.6 mmol/L; and high TG, > 22.6 mmol/L. TC categories were defined according to sample quartiles: Q1, ≤ 6.63 mmol/L; Q2, > 6.63 to ≤ 8.89 mmol/L; Q3, > 8.89 to ≤ 11.75 mmol/L; and Q4, > 11.75 mmol/L. All P values were > 0.05, indicating no statistically significant differences between the categorized lipid groups and their respective reference groups in these categorized analyses.
